# A Multi-Model, Pixel-Native Framework for Automated Computed Tomography Series Labeling and Characterization: Proof-of-Concept Study

**DOI:** 10.2196/93018

**Published:** 2026-09-29

**Authors:** Yutong Wen, Anton Sheahan Quinsten, Cynthia Sabrina Schmidt, Christian Bojahr, Judith Kohnke, Kamyar Arzideh, Sina Warmer, Sebastian Blex, Ann-Christin Jacoby, Max Eberts, Hanna Lehmann, Olivia Barbara Pollok, Mathias Holtkamp, Luca Salhöfer, Lale Umutlu, Michael Forsting, Johannes Haubold, Felix Nensa, Katarzyna Borys, René Hosch

**Affiliations:** 1 Data Integration Center Central IT Department University Hospital Essen Essen Germany; 2 Institute for Artificial Intelligence in Medicine (IKIM) University Hospital Essen Essen Germany; 3 Institute of Diagnostic and Interventional Radiology and Neuroradiology University Hospital Essen Essen Germany; 4 Institute for Transfusion Medicine University Hospital Essen Essen Germany; 5 Center of Sleep and Telemedicine University Hospital Essen - Ruhrlandklinik Essen Germany

**Keywords:** AI orchestration, computed tomography, deep learning, health information interoperability, reconstruction kernel, anatomical region detection, radiology workflow, Digital Imaging and Communications in Medicine–free classification, DICOM-free classification, artificial intelligence, AI

## Abstract

**Background:**

Although Digital Imaging and Communications in Medicine (DICOM) metadata are widely used to manage medical imaging data and support clinical workflows, their suitability as a sole basis for automatic computed tomography (CT) series labeling and characterization is limited. DICOM metadata are frequently inconsistently populated, institution specific, use unregulated private tags, and have variable reliability even within standardized fields. Consequently, automated series selection for downstream AI applications often remains unreliable, necessitating manual curation within clinical workflows.

**Objective:**

This study presents Orchestrate, a modular AI framework for automated orchestration of CT imaging data. By integrating a hierarchy of deep learning models, Orchestrate enables pixel-level classification and routing of CT series and accurate metadata-independent identification of anatomical regions, contrast-enhanced series, and reconstruction kernels, supporting seamless downstream AI integration without manual curation.

**Methods:**

Orchestrate combined 3 pretrained models for anatomical regions, landmarks, and body part classification and 4 newly developed You Only Look Once (YOLO) v8–based models to classify contrast enhancement, recognize reconstruction kernels, and infer laterality. Three datasets were used: an internal development dataset comprising 27,418 CT studies for individual model development, an internal framework evaluation dataset comprising 200 CT studies to assess the complete framework under a simulated real-world scenario, and an external dataset from The Cancer Imaging Archive comprising 100 CT studies. For the internal development data, reference standards were derived from complete and unambiguous DICOM metadata following institutional definitions. For the 2 evaluation datasets, 3 radiographers independently established reference standards as DICOM metadata were not assumed to be complete or consistent. Clinical utility was assessed through cohort selection tasks involving 3 predefined target cases, with 3 radiologists reviewing selection accuracy. The interrater agreement was assessed using the Fleiss κ. The model performance was evaluated using *F*_1_-scores.

**Results:**

DICOM metadata were incomplete in the internal framework evaluation dataset and external dataset, with missing rates of 42.9% (413/963) and 92.9% (105/113) for contrast enhancement, respectively, and a missing reconstruction kernel information rate of 0.4% (4/963) in the internal framework evaluation dataset. During model development, individual models achieved macro–*F*_1_-scores ranging from 0.982 to 0.989. At the framework level, Orchestrate achieved high classification performance across internal (weighted *F*_1_-score ranged from 0.920 to 1.000; macro–*F*_1_-score ranged from 0.879 to 1.000) and external (weighted *F*_1_-score ranged from 0.946 to 1.000; macro–*F*_1_-score ranged from 0.777 to 0.929) cohorts. For clinical use cases, the overall selection accuracy was 97.7% (217/222).

**Conclusions:**

Orchestrate enables automated pixel-based classification, detection, and semantic description of CT series, reducing reliance on manual selection and the risk of inconsistent metadata. By generating standardized semantic content, the framework provides a proof of concept for improving interoperability with clinical systems and supports the reliable, reproducible integration of AI-driven imaging pipelines into clinical workflows.

## Introduction

AI, particularly deep learning, has made significant advances in radiology, supporting a broad spectrum of radiology tasks, including anatomical landmark detection and segmentation [1-12], body composition analysis [13-17], virtual biopsy [18-20], and contrast optimization [21-23]. Despite strong performance in controlled settings, translating these advances into routine clinical practice remains challenging. A key bottleneck is the lack of robust, automated mechanisms for selecting and routing the appropriate image series to the corresponding model, resulting in human-in-the-loop selection and routing [24].

This challenge is particularly evident in computed tomography (CT), where a single CT study may include multiple image series, such as localizers, axial volumes, multi-planar reformats, and diverse reconstructions that vary in kernel and contrast phase. In the current workflow, imaging routing largely depends on Digital Imaging and Communications in Medicine (DICOM) metadata. However, the available metadata fields are frequently incomplete and ambiguous across institutions, which complicates automated routing and the reliable selection of relevant image series for downstream AI tasks [25-27].

These limitations can stem not only from inconsistent metadata entries but also from the underlying DICOM metadata structure and its varied implementation across vendors or institutions. Although these metadata remain the canonical sources of acquisition and protocol parameters, their reliability and reusability for accurately describing series content are limited [28]. Metadata fields are often variably populated, and many are optional (eg, the convolution kernel tag [29]), resulting in inconsistent definitions or mislabeling across manufacturers or institutions for relevant selection parameters, such as contrast media and body part information [25,30-32]. In addition, reliance on nonstandardized free-text descriptors such as series description introduces ambiguity and may be inadequate for identifying the series without visual inspection [30]. Moreover, because DICOM metadata operate at the series or study level, they lack the granularity to express voxel- or pixel-level phenomena, such as the distribution of specific body regions within one CT series or the detection of indwelling devices. Collectively, these limitations undermine standardization and introduce inefficiencies, necessitating manual curation to identify the correct input series for AI processing [30]. Beyond workflow burden, such inconsistencies also limit reproducibility in research, complicate multi-institutional model development, and may compromise clinical reliability when models are inadvertently applied to suboptimal or incorrect series.

To overcome these limitations, we introduce Orchestrate, a modular framework for fully automated classification, detection, and semantic enrichment of CT image series. In contrast to approaches that depend primarily on DICOM metadata, Orchestrate performs coordinated, image-based reasoning through a hierarchy of specialized classifiers and detectors, extracting clinically and technically relevant information directly from the image content. The framework infers key series characteristics (including anatomical coverage, landmarks, contrast enhancement, scan orientation, reconstruction kernel, and laterality), thereby substantially increasing the semantic depth available per CT series. By fusing image-derived content features with existing DICOM metadata, Orchestrate enables robust, scalable, and reliable CT series routing and selection even in the presence of incomplete, inconsistent, or erroneous metadata.

## Methods

### Ethical Considerations

This retrospective study was approved by the Ethics Committee of the University Hospital Essen (approval number 21-10204-BO). The requirement for written informed consent was waived due to the study’s retrospective nature. All data were fully anonymized before being included in the study.

### CT Datasets and Acquisition

This study included 3 CT datasets, each serving a distinct purpose. The internal development dataset, comprising 27,418 CT studies acquired between April 2002 and September 2024 at a single medical center, was used to develop and evaluate the individual models in the framework. Before data retrieval, studies were selected through the institutional Fast Healthcare Interoperability Resources (FHIR) server using predefined query parameters and values (eg, specific reconstruction kernel values such as Br32) corresponding to metadata attributes required for model development such that only studies with the required metadata fields and values were retrieved. Most CT studies (n=26,518, 96.7%) were acquired using Siemens Healthineers scanners, with the remaining studies acquired using Philips scanners (n=285, 1%), GE HealthCare scanners (n=12, 0%), and Canon Medical Systems scanners (n=603, 2.2%). Additional details for each individual model are summarized in Multimedia Appendix 1 [33-36].

The internal framework evaluation dataset comprised 200 CT studies acquired between October 2024 and November 2024 at the same medical center. This dataset was independent of the development dataset, with no patient overlap with the training or validation sets used for any individual model. It was used to simulate a real-world clinical scenario and assess the integrated framework’s performance. Therefore, DICOM metadata in this dataset were not required to be complete or valid, and some metadata fields were missing. The CT scanner manufacturer represented in this dataset was Siemens Healthineers. In addition, an external dataset comprising 100 CT studies randomly selected from a CT colonography dataset, the Cancer Imaging Archive (TCIA) [37], served as an independent external evaluation to assess the framework’s robustness and generalizability under heterogeneous imaging conditions. CT scanner manufacturers represented in this dataset included Siemens Healthineers (70/100, 70% of the studies), GE HealthCare (22/100, 22% of the studies), Toshiba Medical Systems (now Canon Medical Systems; 6/100, 6% of the studies), and Philips (2/100, 2% of the studies).

### Orchestrate Framework Architecture

#### Overview

The Orchestrate pipeline is structured as a modular tree of deep learning models designed to extract comprehensive semantic information directly from image pixels. This framework facilitates the automated classification and detection of CT topograms and series across multiple clinical dimensions, forming the foundation for pixel-native routing, selection, and structured FHIR descriptions. At its core, Orchestrate integrates a hierarchy of multiple You Only Look Once (YOLO) v8–based [38,39] classification models, each tailored to a specific classification task, together with the rapid analysis and processing of image data (RAPID) [40] models summarized in Table 1. Detailed definitions of classes, representative input examples, training protocols, and hyperparameter configurations for each model are provided in Multimedia Appendix 1 [33-36].

**Table 1:** Overview of integrated models in the Orchestrate framework^a^.

Model name	Task	Output classes
View Position	Determines the anatomical plane of the topogram	Lateral and nonlateral
RAPID^b^ Classification	Classifies the anatomical coverage of the topogram	Head, upper extremities, lower extremities, and torso
Body Contrast Enhancement	Differentiates native and contrast enhancement of the body CT^c^ series	Native and contrast enhancement
Brain Contrast Enhancement	Determines native and contrast media series for brain CT	Native and contrast enhancement
Reconstruction Kernel	Identifies reconstruction kernel based on image texture and noise patterns	SK^d^ and HK^e^
RAPID Body Regions	Detects and localizes body regions from the given topogram	Head, abdominal region, thoracic region, and pericardium
RAPID Landmarks	Detects and localizes body landmarks from the given topogram	Lung, heart, spine, liver, kidneys, spleen, stomach, colon, pancreas, brain, and hip

^a^Contrast enhancement refers only to intravenous contrast.

^b^RAPID: rapid analysis and processing of image data.

^c^CT: computed tomography.

^d^SK: soft kernel.

^e^HK: hard kernel.

#### Pipeline Structure

As illustrated in Figure 1 [41], the pipeline is initiated with the view position classifier, which categorizes the anatomical plane of the topograms as lateral or nonlateral. All topograms are subsequently processed by the multi-class RAPID classification model, which assigns them to 1 of the 4 broad categories: head, upper extremities, lower extremities, and torso. In parallel, topograms identified as lateral are routed to the RAPID Landmarks model. If cranial landmarks (brain) are detected on the lateral topogram or the RAPID Classification model independently identifies the lateral topogram as being of the “head” class, the entire study is forwarded to the Brain Contrast Enhancement classifier for further evaluation.

**Figure 1:**
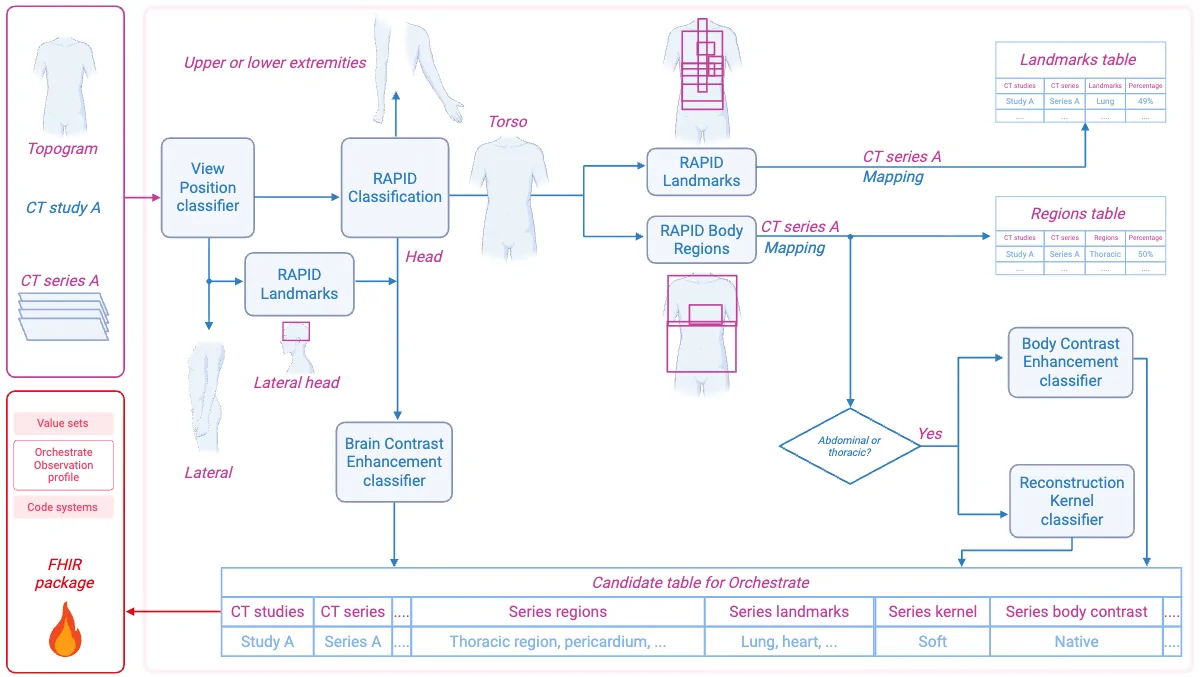
Schematic overview of the complete Orchestrate framework. This overview presents the overall logic of the proposed framework. Computed tomography (CT) studies are analyzed using the corresponding topogram to localize and quantify the proportional coverage of anatomical structures. CT series containing thoracic or abdominal regions are subsequently processed by task-specific classifiers. The processed results are stored in 3 relational database tables: the landmarks and regions table for anatomical coverage and a candidate table aggregating outputs from all Orchestrate models that can support further target cohort selection. The Fast Healthcare Interoperability Resources (FHIR) package provides a downstream semantic mapping layer to support interoperability and is not part of the core Orchestrate logic. Created in BioRender [41]. RAPID: rapid analysis and processing of image data.

For studies with nonlateral topograms classified as “torso,” the RAPID Body Regions and RAPID Landmarks models are executed in parallel to characterize their anatomical location. The RAPID Body Regions model detects anatomical subregions (eg, thoracic region or abdominal region; Table 1) using bounding boxes on the topogram. In contrast, the RAPID Landmarks model identifies key skeletal and soft-tissue landmarks (Table 1). Anatomical regions and landmarks in both RAPID models were defined according to the Body and Organ Analysis (BOA) [13] and TotalSegmentator [12] tools. Thereafter, each axial CT series is geometrically mapped to the corresponding topogram using DICOM-reported slice coordinates and the scanning direction. The intersection between mapped slice locations and detected anatomical regions or landmarks enables estimation of the anatomical series coverage. Subsequently, each CT series containing the abdominal or thoracic region is processed using contrast and reconstruction kernel classifiers, as defined in Table 1.

#### Automated Series Exclusion Criteria

Orchestrate incorporates an automated filtering module that excludes CT series unsuitable for semantic processing. Exclusion criteria include nonaxial images, invalid modality (modalities other than CT), nonimage content such as dose reports, or content with additional segmentation masks or annotations. Series with insufficient anatomical information, such as those that do not contain any detectable region or landmark after topogram mapping, are also excluded. Moreover, studies with missing, corrupted, or anatomically uninterpretable topograms are excluded from further processing. All exclusions are automatically recorded in the orchestrate database.

#### Semantic Aggregation

All model outputs are aggregated into a unified, structured semantic description for each CT series. To demonstrate interoperability, an initial mapping of the stored data to the FHIR server is provided. The FHIR profile presented in this study serves as an illustrative reference rather than a finalized standard.

### Reference Standard

For the internal development dataset, reference standards for the individual model components were derived from the corresponding DICOM metadata attributes used as predefined query parameters for study selection and were used for model training, validation, and internal testing.

In contrast, the internal framework evaluation dataset was retrieved without metadata-based selection, and the DICOM metadata were not required to be complete or consistently valid. Therefore, 3 experienced radiographers (over 20, 18, and 8 years of experience) with expertise in CT acquisition protocols and reconstruction parameters independently reviewed all the CT series processed through the complete orchestration framework for the internal framework evaluation dataset and the external dataset from TCIA to generate corresponding reference standards. To minimize potential bias, the reviewers were blinded to the dataset origin (internal or external), scanner information, and DICOM metadata of each series. Given the objective nature of the view position classification task (lateral vs nonlateral topogram), a medical AI data scientist assigned reference standards under the guidance of a radiologist with over 3 years of experience. For the clinically applicable target scenarios requiring cohort-specific anatomical information, 3 experienced radiologists (over 5, 2, and 2 years of experience) additionally reviewed all the selected CT series for each target cohort to determine the anatomical structures covered and assess contrast enhancement. Reconstruction kernels were evaluated by the 3 radiographers described above.

### Evaluation Framework and Statistical Analysis

Patient age was reported as means and SDs or medians with IQRs depending on the normality of the age distribution, assessed using the Shapiro-Wilk test [43] for smaller datasets (n<5000) or the Anderson-Darling test [44]. For the external dataset, patient age and sex information were extracted directly from the corresponding DICOM metadata.

Individual model performance was evaluated on their independent test sets using accuracy, precision, recall, *F*_1_-scores, and the area under the receiver operating characteristic curve with 95% CIs, which were estimated using bootstrapping with 1000 iterations. Interrater agreement was assessed using the Fleiss κ for agreement among multiple raters and the Cohen κ for pairwise agreement. The performance of the completed framework was evaluated using *F*_1_-scores with 95% CIs. Statistical analyses were conducted using the Python packages SciPy [45], scikit-learn [46], and statsmodels [42].

### Clinically Applicable Targets

To evaluate the performance and clinical utility of the Orchestrate framework, 3 clinically relevant orchestration targets were defined based on anatomical coverage, contrast enhancement, and reconstruction kernel (Table 2), each reflecting typical diagnostic CT imaging scenarios that potentially involve AI vendors. The specified craniocaudal coverage percentage presented in Table 2 was configurable, and the values served as examples that can be adapted to local clinical practice.

**Table 2:** Definition and anatomical scope of target cases and their relevance to clinical AI applications.

Target case	Anatomical coverage	AI application context
Contrast-enhanced liver CT^a^ with smooth reconstruction kernel	These CT series provide complete craniocaudal coverage of the liver. To differentiate liver-focused CT examinations for this target case from general abdominal or thoracic series, which may also include the entire liver, the anatomical coverage is further constrained based on the craniocaudal anatomical coverage. Specifically, liver CT series for this case were required to include less than 50% craniocaudal coverage of both the abdominal and thoracic regions. This threshold was treated as a tunable parameter to accommodate protocol variability across institutions.	This target represents a typical abdominal imaging task, where precise identification of the contrast enhancement and reconstruction type is crucial for downstream AI applications, including lesion detection and liver volumetry.
Contrast-enhanced thoracic CT with hard reconstruction kernel	The topogram of these studies should demonstrate complete craniocaudal coverage of the thorax. The area of interest for the thoracic CT should include the entire lung region [47]. Similar to the constraint in the previous target case, the thoracic CT for this case must encompass the near-complete thoracic region, with abdominal coverage limited to less than 30% of the total abdominal extent in the z-axis direction.	This is a frequent setting in cardiovascular and oncological imaging, requiring precise recognition of contrast enhancement and high-resolution lung kernel reconstructions.
Whole-body CT with smooth reconstruction kernel	Both the scout topogram and CT series must demonstrate at least complete craniocaudal coverage of the thoracic and abdominal regions.	This can be used in trauma or oncology staging, requiring full-body coverage and a preference for soft-tissue kernel reconstructions to ensure compatibility with general-purpose AI algorithms.

^a^CT: computed tomography.

## Results

### Patient Characteristics

The internal development dataset included 17,362 unique patients (n=7852, 45.2% female). Patient age was not normally distributed (Anderson-Darling test *A*^2^=439.600), and the median age was 63 (IQR 52-72) years. The internal framework evaluation dataset comprised 185 unique patients (n=100, 54.1% male). Patient age was also nonnormally distributed (Shapiro-Wilk *W*=0.957; *P*<.001) and was summarized accordingly (median 63.50, IQR 51-72.25 years).

The external dataset included 99 unique patients (n=47, 47.5% female), of whom 16 (16.2%) were missing age information and 1 (1%) had an invalid age. The ages of the remaining 82.8% (82/99) of the patients were not normally distributed (Shapiro-Wilk *W*=0.787; *P*<.001), with a median of 56 (IQR 52-60.75) years.

### Individual Model Performance

All individual models demonstrated high performance on their respective independent test sets (Table 3).

All performance metrics are reported with 95% CIs, with accuracy and macroaverage recall ranging from 0.983 to 0.989, macroaverage precision ranging from 0.978 to 0.989, macroaverage *F*_1_-score ranging from 0.982 to 0.989, and area under the receiver operating characteristic curve ranging from 0.995 to 0.998.

**Table 3:** Classification performance of individual models.

	View Position	Body Contrast Enhancement	Brain Contrast Enhancement	Reconstruction Kernel
Accuracy (95% CI)	0.989 (0.980-0.996)	0.985 (0.982-0.989)	0.983 (0.978-0.988)	0.984 (0.979-0.989)
Precision (95% CI)	0.989 (0.979-0.996)	0.978 (0.972-0.983)	0.983 (0.978-0.988)	0.984 (0.980-0.989)
Recall (95% CI)	0.989 (0.980-0.997)	0.986 (0.982-0.989)	0.983 (0.978-0.988)	0.983 (0.978-0.988)
*F*_1_-score (95% CI)	0.989 (0.980-0.996)	0.982 (0.977-0.986)	0.983 (0.978-0.988)	0.984 (0.979-0.988)
ROC AUC^a^ (95% CI)	0.995 (0.987-1.000)	0.998 (0.997-0.999)	0.997 (0.996-0.999)	0.998 (0.996-0.999)

^a^ROC AUC: area under the receiver operating characteristic curve.

### Orchestrate Evaluation

For the internal framework evaluation dataset, the framework received 200 studies with 2003 series. During framework processing, of the 2003 series, 200 (10.0%) were identified as topograms, 309 (15.4%) contained nonimage data, 214 (10.7%) were positron emission tomography components, 241 (12.0%) were nonaxial series, and 23 (1.1%) contained segmentations. The remaining 1016 series were forwarded to the respective models, and of these series, 53 (5.2%) did not cover any detectable body regions, whereas 963 (94.8%) were successfully processed. Of the 963 successfully processed series, 225 (23.4%) underwent brain contrast classification, and 738 (76.6%) underwent further reconstruction kernel and body contrast enhancement classifications, and their results were stored in the database’s candidate table and subsequently displayed to radiographers, radiologists, and medical imaging AI researchers through the Orchestrate viewer, as shown in Figure 2. Among the successfully processed 963 series, reconstruction kernel and contrast enhancement information was missing in 4 (0.4%) and 413 (42.9%), respectively. Of the 550 series with available contrast information, 292 (53.1%) indicated only the presence of contrast enhancement without specifying intravenous or gastrointestinal contrast enhancement.

The external dataset comprised 100 studies with 202 topograms, with each study acquired in both prone and supine positions. During the framework processing, 1.5% (3/202) of the topograms could not be loaded because of corrupted DICOM files or inconsistencies between DICOM header and pixel data. Of the remaining 199 topograms, 24 (12.1%) were classified as lateral topograms without head coverage. Of the topograms classified as nonlateral, body region detection failed in 35.4% (62/175). The remaining 64.6% (113/175) of the topograms, with 113 CT series, were successfully processed through the framework for further reconstruction kernel and body contrast enhancement classifications, and results were stored in the database. Reconstruction kernel information was available in the DICOM metadata for all series. Contrast enhancement information was available for only 7.1% (8/113) of the series without specifying intravenous or gastrointestinal contrast enhancement. For the remaining 92.9% (105/113) of the series, contrast enhancement information was missing from the metadata.

Three radiographers independently reviewed all processed internal and external series to generate the reference standards. Interrater agreement for reconstruction kernel, body contrast enhancement, and brain contrast enhancement was assessed using the Fleiss κ, with values of 0.931 (95% CI 0.910-0.950), 0.936 (95% CI 0.915-0.956), and 0.958 (95% CI 0.918-0.991), respectively, indicating good agreement among the 3 radiographers. Additional pairwise agreement analyses can be found in Multimedia Appendix 2 [13,48-50]. The final reference standards for each series were determined through majority agreement among the 3 radiographers.

Table 4 presents Orchestrate’s overall performance using weighted-average and macroaverage *F*_1_-scores with 95% CIs.

**Figure 2:**
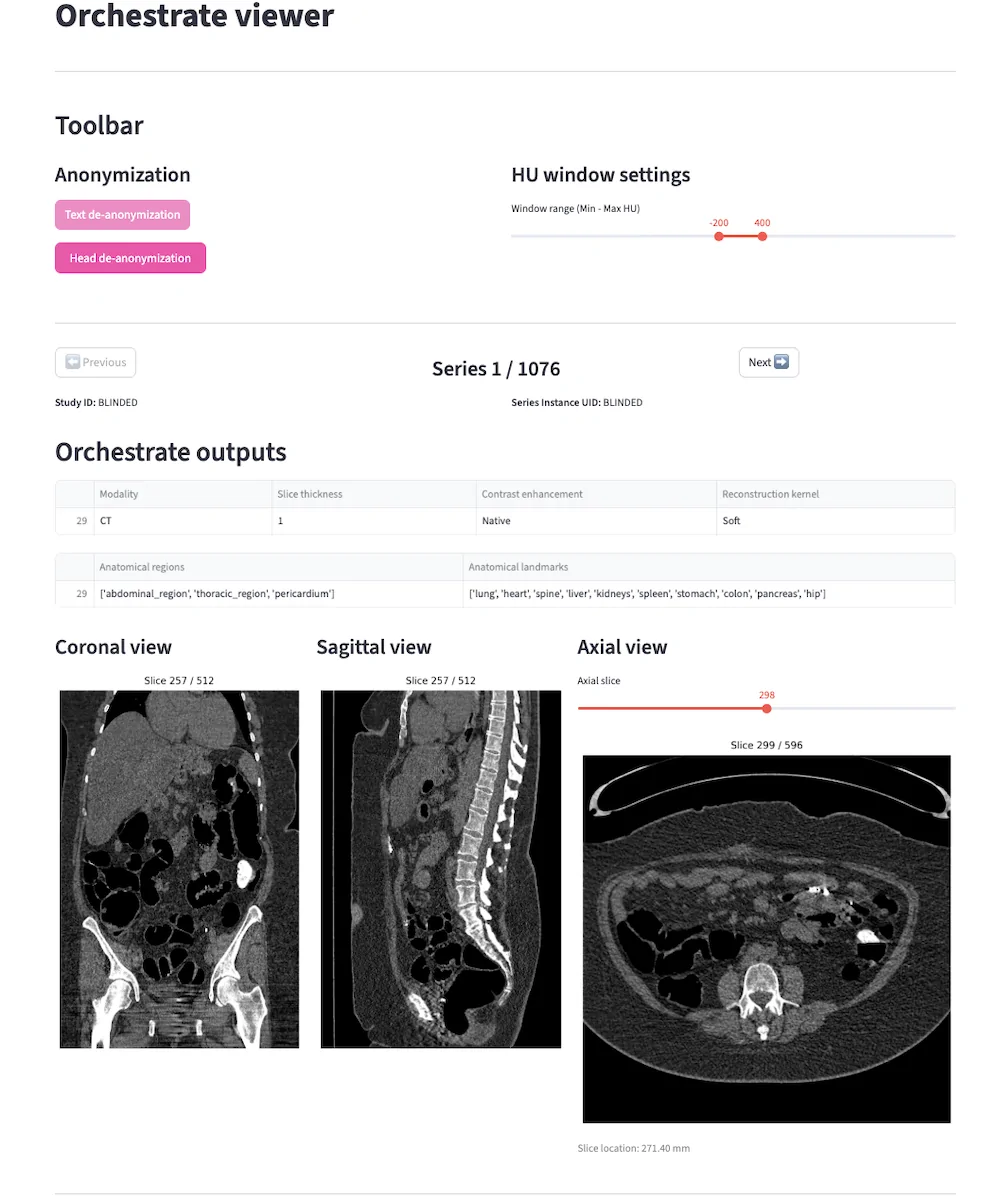
Interactive Orchestrate dashboard. The dashboard visualizes results from automated processing in the Orchestrate pipeline, including thickness, contrast enhancement, reconstruction kernel, anatomical region distribution, and detected anatomical landmarks, and incorporates insights from radiologists and researchers to serve diverse user needs. The dashboard provides clinical-grade validation tools for clinicians, including adjustable Hounsfield unit (HU) windowing controls and a comprehensive axial viewer with a navigator, which allows users to scroll through all slices and enables rapid, direct manual verification when they require more detail from the processing results. On the basis of the researchers’ feedback, the dashboard also includes midcoronal and midsagittal cross-sectional views. These planes provide an intuitive anatomical overview for users without extensive medical imaging expertise, as well as for those who prefer alternatives to traditional axial slice navigation.

**Table 4:** Comparison of model performance between the internal framework evaluation dataset and the external dataset using weighted and macro–*F*_1_-scores.

Model name and metric		Internal framework evaluation dataset	External dataset
**View Position**			
	Weighted *F*_1_-score (95% CI)	1.000 (1.000-1.000)	0.970 (0.944-0.990)
	Macro–*F*_1_-score (95% CI)	1.000 (1.000-1.000)	0.929 (0.866-0.977)
**Reconstruction Kernel**			
	Weighted *F*_1_-score (95% CI)	0.986 (0.977-0.993)	1.000 (1.000-1.000)
	Macro–*F*_1_-score (95% CI)	0.986 (0.977-0.993)	N/A^a,b^
**Brain Contrast Enhancement**			
	Weighted *F*_1_-score (95% CI)	0.920 (0.876-0.954)	N/A^c^
	Macro–*F*_1_-score (95% CI)	0.879 (0.814-0.929)	N/A^c^
**Body Contrast Enhancement**			
	Weighted *F*_1_-score (95% CI)	0.989 (0.981-0.996)	0.946 (0.909-0.979)
	Macro–*F*_1_-score (95% CI)	0.983 (0.970-0.994)	0.777 (0.582-0.905)

^a^N/A: not applicable.

^b^Only 1 class in the reference standard.

^c^Head not included.

The View Position classifier was assessed using topograms, and all remaining models were evaluated on CT series. The Brain Contrast Enhancement classifier was not evaluated in the external cohorts because the head was not included in the external CT scans. For the Reconstruction Kernel classifier, only the weighted-average *F*_1_-score was reported because all external scans belonged to a single reference standard class (soft kernel). All models achieved good performance across both internal and external data. In the internal framework evaluation dataset, weighted-average *F*_1_-scores ranged from 0.920 to 1.000, whereas macroaverage *F*_1_-scores ranged from 0.879 to 1.000. In the external dataset, macroaverage *F*_1_-scores ranged from 0.777 to 0.929, and weighted-average *F*_1_-scores ranged from 0.946 to 1.000. Model-specific error analyses are summarized in Multimedia Appendix 2 [13,48-50]. In addition, Orchestrate achieved a total processing time for the internal framework evaluation dataset and external dataset of 3.066 seconds per CT study on a workstation equipped with an NVIDIA RTX A6000 graphics processing unit (49140-MiB memory) and 1-TiB system memory. Additional computational details and system requirements are provided in Multimedia Appendix 1 [33-36].

### Orchestration Use Case Evaluation

Three radiologists (contrast enhancement and anatomical coverage) and 3 radiographers (reconstruction kernel) independently reviewed all selected series for each use case. Table 5 summarizes the review results from human experts and the accuracy of each target use case.

**Table 5:** Expert validation of target use cases based on anatomical coverage, contrast enhancement, and reconstruction kernel criteria with accuracy assessment.

Target use case	Series, n	Anatomical coverage and contrast enhancement information	Reconstruction Kernel model	Accuracy, n/N (%)
Contrast-enhanced liver CT^a^ and soft kernel	35	35 matched	35 matched	35/35 (100)
Contrast-enhanced thorax CT and hard kernel	134	1 mismatched and 133 matched	3 mismatched and 131 matched	130/134 (97.0)
Whole-body CT and soft kernel	53	53 matched	1 mismatched and 52 matched	52/53 (98.1)

^a^CT: computed tomography.

All selected series in the contrast-enhanced liver CT with soft kernel use case met the predefined selection criteria. In the whole-body CT with soft kernel use case, 1.9% (1/53) of the series had a misclassified reconstruction kernel, whereas in the contrast-enhanced thorax CT with hard kernel use case, 3.0% (4/134) of the series in total did not match the defined criteria. The corresponding selection accuracies were 100% (35/35), 98.1% (52/53), and 97.0% (130/134), respectively. Across all 3 use cases, 2.3% (5/222) of the selected series did not meet the corresponding selection criteria, resulting in an overall accuracy of 97.7% (217/222).

The anatomical coverage thresholds applied in the target set can be adapted according to the requirements of the given application. For example, for contrast-enhanced thorax CT, a broader range of abdominal coverage may be acceptable when additional abdominal anatomy does not interfere with the intended analysis, and a more restrictive threshold may be preferred for applications such as lung tumor detection. Figure 3 illustrates this flexibility using example middle coronal slices from 2 contrast-enhanced thorax CT series with different degrees of abdominal coverage together with their corresponding topograms and outputs from RAPID Body Regions.

**Figure 3:**
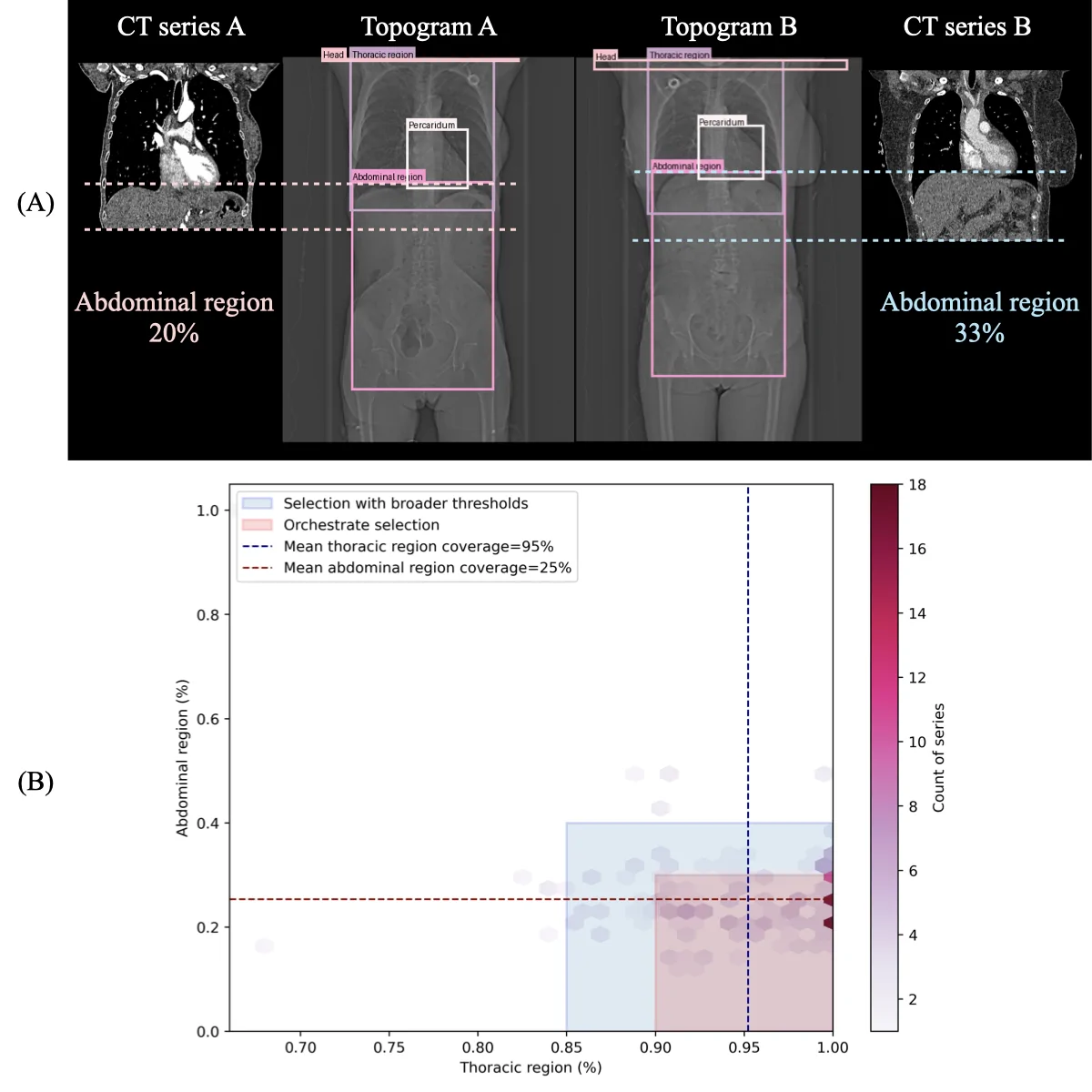
Visualization of abdominal and thoracic coverage thresholds for contrast-enhanced thorax computed tomography (CT) selection. (A) Example coronal images in the upper panel show CT series with different degrees of abdominal coverage together with their corresponding topograms and bounding boxes for body regions. The contrast of the topograms shown in this figure was adjusted to improve visualization of anatomical details. The plot (B) demonstrates the distribution of contrast-enhanced thorax CTs that radiologists considered suitable based on their relative thoracic and abdominal coverage. The red rectangle indicates the predefined coverage thresholds applied for the target use case in this study, whereas the blue rectangle indicates a scenario using broader selection thresholds. The adjustable threshold values serve as operational parameters for automated selection rather than universal clinical criteria.

To further illustrate the effect of the predefined thresholds on series selection, 3 radiologists independently reviewed the complete candidate series pool and identified contrast-enhanced thorax CTs that they considered suitable for thoracic CT analysis. All candidate series had previously been classified as having a hard reconstruction kernel based on majority agreement among 3 radiographers. Finally, 215 series were considered eligible based on the majority agreement among radiologists. Figure 3 demonstrates the distribution of these series with relative abdominal and thoracic coverage.

The predefined threshold for the target use case restricted the selection to the region indicated by the red rectangle in Figure 3. The thresholds were introduced in this study to demonstrate how automated selection can be operationalized and should not be interpreted as universal clinical criteria. Therefore, series outside this region are not necessarily incorrectly selected or clinically unsuitable for thoracic CT analysis, and adjusting the thresholds would allow us to select more series. For example, expanding the criteria to allow for less than 40% abdominal region and more than 85% thoracic region would include most of the series considered suitable by the radiologists (blue rectangle in Figure 3).

## Discussion

### Principal Results

This study introduces Orchestrate, a modular, pixel-native AI orchestration pipeline designed to rapidly automate CT series classification, image routing, and structured metadata generation without relying entirely on inconsistent DICOM metadata, a weakness highlighted both in previous works, such as undefined private tags [25,51], false entries in approximately 15% of images [52], and missing body region information in 32% of series [25], and in the analysis across both the internal framework evaluation dataset and external dataset in this study, which revealed that more than 42% of contrast enhancement information was missing for each dataset. Even when this information is available, the route of contrast administration (intravenous or gastrointestinal) is not consistently specified. In addition, reconstruction kernel information was missing in fewer than 1% of series (4/963, 0.4%) in the internal framework evaluation dataset.

For the internal framework evaluation dataset, Orchestrate demonstrated high performance in body contrast enhancement recognition, reconstruction kernel–type identification, and laterality determination, with all macro– and weighted *F*_1_-scores larger than or equal to 0.983, enabling robust and automated characterization of CT image series in real-world clinical data, reducing dependence on manual curation, and supporting consistent preparation of imaging studies for clinical interpretation and AI integration. These outputs provide immediate feedback on series for specific clinical or research requirements. Moreover, the high weighted *F*_1_-scores across different tasks in the external dataset indicate that the framework maintained consistent performance when applied to data acquired from the other medical centers, particularly for the View Position and Body Contrast Enhancement classifiers. However, external evaluation of the remaining models was limited by the availability and class composition of the external dataset.

Beyond quantitative performance, which confirms technical accuracy, the true value of Orchestrate lies in its clinical utility and research applications. To assess Orchestrate’s clinical utility, the system was evaluated across 3 clinically relevant orchestration targets, consistently demonstrating high performance. All automatically selected series of contrast-enhanced liver CT with a soft kernel met the predefined selection criteria. Moreover, whole-body CT with a soft kernel and contrast-enhanced thorax CT with a hard kernel achieved selection accuracy of 98.1% (52/53) and 97.0% (130/134), respectively. These results confirm that Orchestrate reliably replicates human-level classification decisions under variable real-world conditions.

To facilitate seamless integration and interoperable use of Orchestrate outputs within existing clinical workflows, enabling institutions to automate metadata enrichment, quality control, protocol compliance checks, and secure data export, an initial FHIR [53] profile was developed as a proof of concept to standardize the representation of the pixelwise results, anatomical coverage information, and associated CT metadata for prospective integrations. The implementation details, including definitions, value sets, code systems, and examples, are provided in Multimedia Appendix 3 [53,54]. This instantiation is not intended as a finalized representation, and future work may refine the profile for greater precision.

Beyond the study’s primary purpose, Orchestrate also enables a practical, automated head anonymization approach for CT series as a secondary clinical application prior to data sharing or research use (the detailed methodology and validation are provided in Multimedia Appendix 4), which prevents the extraction of reproducible facial information from CT series [55]. This anonymization capability demonstrates Orchestrate’s broader utility and highlights its potential to preserve privacy in clinical imaging workflows.

### Comparison With Prior Work

Previous approaches have addressed CT contrast characterization using different task formulations and input representations. FALCON [48] primarily focuses on binary classification of the presence or absence of intravenous contrast using a single axial slice. BOA-Contrast [49] performs more detailed contrast characterization using Hounsfield unit features from relevant body structures produced by the BOA segmentation framework [13]. The Body Contrast Enhancement classifier in Orchestrate uses 4 selected axial slices combined into a single PNG image for binary classification of intravenous contrast enhancement.

To provide a quantitative comparison, we evaluated the Body Contrast Enhancement classifier in Orchestrate against BOA-Contrast and FALCON using the same internal framework evaluation and external datasets (Multimedia Appendix 2 [13,48-50]). The Body Contrast Enhancement classifier achieved slightly higher *F*_1_-scores in both cohorts. Pairwise prediction agreement among the 3 approaches ranged from 0.667 to 0.806, indicating varying degrees of similarity in the models’ predictions and highlighting the challenging nature of contrast enhancement classification.

Beyond individual model performance, Orchestrate’s main distinction is integrating multiple CT characterization tasks within a lightweight, image-based framework, which provides complementary benefits across different clinical roles. Physicians, particularly radiologists and radiographers, can assess series-level descriptors and anatomical completeness, thereby promptly identifying inappropriate series and minimizing the need for manual review. In addition, researchers could use the standardized output to efficiently normalize metadata and automate cohort selection based on anatomical coverage and acquisition-phase standards. Beyond research applications, this system could also be used to perform in-place checks and raise status alerts when missing acquisitions or mismatches are detected, enhancing overall data quality. Collectively, these capabilities highlight Orchestrate’s value for real-world radiology operations.

### Limitations and Future Work

Several limitations should be acknowledged. First, the current implementation is designed for axial CT series, where sagittal and coronal reconstructions are not yet included because of orientation inconsistencies in DICOM metadata, and lateral topogram analysis is currently limited to head examination. A future direction is to incorporate orientation correction methods and extend topogram analysis to additional projections. Furthermore, Orchestrate is currently CT specific. Extension to other modalities, such as magnetic resonance imaging, will require adaptation to modality-specific image representations. For example, processing 3D magnetic resonance imaging localizers may benefit from segmentation-based models (eg, those used in automated field of view planning [56]) to process localizers and extend this approach to other image types in the future.

Second, current contrast enhancement models detect the presence or absence of intravenous contrast enhancement but do not differentiate individual enhancement phases or gastrointestinal contrast. This limitation is primarily attributable to 2 factors. First, the CT series can contain mixed contrast phases, such as arterial-venous blends or native-urophase mixtures, which reduce the clarity of contrast phase boundaries and limit the formation of a clean phase-specific ground truth. In addition, the lightweight, computationally efficient [57] architecture used in Orchestrate requires each 3D CT volume to be represented by selected slices combined in a single PNG image as an input. Although this approach improves computational efficiency, these slices may not always capture anatomical regions where intravenous enhancement is most apparent, potentially limiting the information available to the model. While more complex automatic architectures can offer greater granularity, they cannot fully solve this limitation [49]. A potential future approach is to use the contrast classifier on different anatomical regions, enabling more contextual contrast evaluation within localized anatomical subsets.

Third, the internal development dataset was constructed through targeted retrieval using predefined DICOM metadata values and attributes, and cases with missing or ambiguous DICOM metadata required to define the corresponding model classes were not included. However, human experts did not independently verify all these DICOM-derived labels, and residual labeling errors cannot be completely ruled out.

Finally, although the external TCIA evaluation introduced heterogeneity, further prospective multicenter validation is needed to assess generalizability across broader clinical environments. Dedicated external evaluation was also not performed for low-dose acquisitions, motion or metal artifacts, or systematically incomplete CT series. Future studies should therefore assess these conditions explicitly and investigate whether institution-specific preprocessing or fine-tuning with representative local data benefits deployment across institutions.

### Conclusions

Orchestrate demonstrates the feasibility of a modular, content-based AI pipeline for rapid and accurate CT series characterization that overcomes limitations posed by incomplete or inconsistent DICOM metadata and enables image-based series characterization. By combining pixel-derived semantics with existing DICOM-based workflows, Orchestrate provides a reliable foundation for automated imaging workflows and streamlined data preprocessing in downstream AI applications.

## Appendix

Multimedia Appendix 1 Data and model development details.

Multimedia Appendix 2 Comparative evaluation and error analysis.

Multimedia Appendix 3 Potential Fast Healthcare Interoperability Resources (FHIR) profile.

Multimedia Appendix 4 Head region exclusion for anonymization.

## Abbreviations

BOA: Body and Organ Analysis
CT: computed tomography
DICOM: Digital Imaging and Communications in Medicine
FHIR: Fast Healthcare Interoperability Resources
RAPID: rapid analysis and processing of image data
TCIA: the Cancer Imaging Archive
YOLO: You Only Look Once

## Data Availability

The dataset used in this study is not publicly available. Individuals or academic organizations interested in using this dataset must submit a detailed request to Data-Governance@uk-essen.de, which will be reviewed case by case. The source code will be publicly available on GitHub [58] after the paper is accepted.
