# Supplementary material for: A Multi-Model, Pixel-Native Framework for Automated Computed Tomography Series Labeling and Characterization: Proof-of-Concept Study
Source: JMIR Med Inform. 2026 Sep 29;14:e93018. doi: 10.2196/93018 (PMC13662436; doi:10.2196/93018)
Supplement: Supplementary file 1 [file medinform_v14i1e93018_app1.docx]

# Data and model development details

## A.1 Dataset Composition and Splits

Table A1 summarizes the internal development datasets for each model. We used a patient-level split, resulting in an approximate distribution of 70%, 15%, and 15% for the training, validation, and test sets, respectively. The data were automatically anonymized during the data download phase.

Table A1. Characteristics of datasets.

| **Model name** | **Class** | **Training** | **Validation** | **Test** | **Total** |
| --- | --- | --- | --- | --- | --- |
| **View Position** | Lateral | 1306 | 263 | 277 | 1846 |
|  | Non Lateral | 1307 | 276 | 263 | 1846 |
|  | **Total** | 2613 | 539 | 540 | 3692 |
| **Body Contrast Enhancement** | CE | 16,480 | 3736 | 3920 | 24,136 |
|  | Native | 6791 | 1396 | 1466 | 9653 |
|  | **Total** | 23,271 | 5132 | 5386 | 33,789 |
| **Brain Contrast Enhancement** | CE | 6310 | 1240 | 1428 | 8978 |
|  | Native | 5951 | 1284 | 1242 | 8477 |
|  | **Total** | 12,261 | 2524 | 2670 | 17,455 |
| **Reconstruction Kernel** | HK | 5952 | 1327 | 1286 | 8565 |
|  | SK | 7642 | 1551 | 1596 | 10,789 |
|  | **Total** | 13,594 | 2878 | 2882 | 19,354 |

## A.2 Definition of soft and hard kernels

Reconstruction kernels are mathematical algorithms used during CT image reconstruction. They can determine the balance between resolution and pixel noise, further modify image sharpness, and adjust image quality [1]. These algorithms can be divided into two groups: hard/high-resolution algorithms, suitable for applications such as bone imaging that operate in the presence of high image noise, and soft algorithms, with lower image noise, that provide good depiction of small density differences in low-contrast settings for applications such as soft tissue imaging [2]. In this study, we classified reconstruction kernels into two categories (Table A2) and validated these categories with a CT radiographer with 20 years of experience.

Table A2. Definition of soft and hard kernels.

| Soft Kernel | I26, I31, I40, I41,  Br32, Br34, Br36, Br38, Br44,  B25, B26, B31, B41, B45, B20, B35, B40, B46,  Bf37,Bv36, Bv38, Bv40, Bv44,  J30, J40,  H20, H31,Hr32, Hr35, Hr36, Hr38, Hr40, Hr44, Hf40,  Qr36, Qr40 |
| --- | --- |
| Hard Kernel | I70,  Br52, Br56, Br59, Br60, Br62, Br64, Br69, Br89,  B60, B70, B75, B80,  Bl57, Bl60, Bl64, Bv59,  J70,  H60, H70, Hr56, Hr59, Hr60, Hr64, Hr68, Hr69,  U90 |

Our kernel categorization aligns with established practices in CT imaging, where soft kernels (such as B30f) are selected for routine soft-tissue evaluation, while hard kernels (such as B50f and B70f) are employed when high-resolution detail is required [3,4].

## A.3 Representative Training Examples

This section presents representative training examples for the four models. All models operate on 2D inputs, and some required multiple slices to form one training sample. We therefore provide examples for each model to illustrate their inputs, along with their corresponding reference standard in Figure A1.

Figure A1. Representative input examples for the four models. The left column indicates the model name, while the remaining two columns show representative model inputs for the two classes.

## A.4 Training Hyperparameters and Tuning

All models were initialized using the pretrained YOLOv8 weights (yolov8x-cls.pt). The initial learning rate (lr0) for each model was determined via a GridSearch over the values [1e-4, 3e-4, 1e-3, 3e-3, 1e-2]. Table A3 summarizes the selected learning rates for the final training of each model. Early stopping was applied to reduce overfitting, with patience thresholds tuned separately for each model (Table A3).

Table A3. Summary of model training hyperparameters and outcomes.

| **Model name** | **lr0** | **patience** | **Epoch** | **Best Epoch** | **Training time** |
| --- | --- | --- | --- | --- | --- |
| View Position | 0.003 | 5 | 15 | 10 | 0.153 hour |
| Body Contrast Enhancement | 0.003 | 10 | 37 | 27 | 3.325 hour |
| Brain Contrast Enhancement | 0.003 | 5 | 17 | 12 | 0.787 hour |
| Reconstruction Kernel | 0.001 | 5 | 17 | 12 | 0.871 hour |

Figure A2 shows the training and validation loss curves to improve transparency and reproducibility, with early stopping based on validation performance.

Figure A2. Training and validation loss curves for all four models.

## A.5 Orchestrate Pre-processing

All data volumes were converted to 640x640 PNG images in the Orchestrate framework. Different CT intensity windows were applied according to each model's preprocessing requirements. Table A4 summarizes the model-specific lower and upper Hounsfield Unit (HU) bounds used for each model. Intensities outside the specified range were clipped to the corresponding bounds, after which the values were linearly normalized.

Table A4. Summary of preprocessing parameters.

| **Model name** | **CT intensity window (HU)** | **Slices per PNG Image** |
| --- | --- | --- |
| View Position | [-1000, 3000] | 1 |
| RAPID Classification | [-1000, 3000] | 1 |
| Body Contrast Enhancement | [-200, 400] | 4 |
| Brain Contrast Enhancement | Volume min – max | 4 |
| Reconstruction Kernel | Volume min – max | 1 |
| RAPID Body Regions | [-1000, 3000] | 1 |
| RAPID Landmarks | [-1000, 3000] | 1 |

## A.6 Computational Details and Requirements

To ensure reproducible pipeline deployment, we provide comprehensive computational specifications. All experiments were conducted on a single workstation. Table A5 presents the pipeline's hardware configuration and processing performance. GPU utilization was continuously monitored using nvidia-smi with a 1-second sampling interval. The number of parallel processes can be adjusted to increase throughput.

Table A5.Computational specifications and processing performance of Orchestrate.

| GPU | NVIDIA RTX A6000 |
| --- | --- |
| GPU Memory | 49140 MiB |
| CPU | AMD EPYC 7402 24-Core Processor |
| RAM | 1.0Ti |
| CUDA | Version 12.0 |
| Number of processes | 10 (adjustable) |
| Total processing time | 919.819 seconds |
| Total processing time / Total number of studies | 3.066 seconds /study |
| Average GPU Utilization | 20.0% |

## References

1[.](https://www.zotero.org/google-docs/?N7sNst)	Schaller S, Wildberger JE, Raupach R, Niethammer M, Klingenbeck-Regn K, Flohr T. Spatial domain filtering for fast modification of the tradeoff between image sharpness and pixel noise in computed tomography. IEEE Trans Med Imaging 2003 July;22(7):846–853. doi: 10.1109/TMI.2003.815073

2[.](https://www.zotero.org/google-docs/?N7sNst)	Riemer A. Computertomografie für MTR/RT: inklusive Herz-CT. 4th ed. Stuttgart: Georg Thieme Verlag KG; 2025. p. b000000820. doi: 10.1055/b000000820ISBN:978-3-13-245241-1

3[.](https://www.zotero.org/google-docs/?N7sNst)	Krishnan AR, Xu K, Li T, Gao C, Remedios LW, Kanakaraj P, Lee HH, Bao S, Sandler KL, Maldonado F, Išgum I, Landman BA. Inter-vendor harmonization of CT reconstruction kernels using unpaired image translation. Proc SPIE-- Int Soc Opt Eng 2024 Feb;12926:129261D. PMID:39268356

4[.](https://www.zotero.org/google-docs/?N7sNst)	Peters AA, Christe A, von Stackelberg O, Pohl M, Kauczor H-U, Heußel CP, Wielpütz MO, Ebner L. “Will I change nodule management recommendations if I change my CAD system?”-impact of volumetric deviation between different CAD systems on lesion management. Eur Radiol 2023 Aug;33(8):5568–5577. PMID:36894752
