# Supplementary material for: A Multi-Model, Pixel-Native Framework for Automated Computed Tomography Series Labeling and Characterization: Proof-of-Concept Study
Source: JMIR Med Inform. 2026 Sep 29;14:e93018. doi: 10.2196/93018 (PMC13662436; doi:10.2196/93018)
Supplement: Supplementary file 2 [file medinform_v14i1e93018_app2.docx]

# Comparative evaluation and error analysis

## B.1 Benchmark Models and Comparative Evaluation

To enhance clarity and facilitate comparison with previous studies, the “Body Contrast Enhancement classifier” is referred to as “Orchestrate-Contrast” exclusively in this Section B.1.

### B.1.1 Architecture benchmark

To quantitatively assess the influence of architecture selection, we additionally evaluated Orchestrate-Contrast on the test set of the internal development dataset, which exhibited the lowest F1 performance with YOLOv8, using the newest YOLO26 from 2026 under the same experimental conditions. The two architectures demonstrated nearly identical predictive performance (Table B1.1). Recall and ROC AUC remained unchanged, while differences in the remaining metrics were approximately 0.001 with substantial overlap in 95% CI. These results indicate that the observed performance differences between YOLOv8 and YOLO26 are negligible. However, because this architecture benchmark was limited to Orchestrate-Contrast, this finding should be interpreted within the scope of this classification task and was not independently assessed for the other models.

Table B1.1. Quantitative comparison of YOLOv8 and YOLO26 architectures

| **Metrics** | **YOLOv8** | **YOLO26** |
| --- | --- | --- |
| Accuracy (95% CI) | 0.985 (0.982-0.989) | 0.986 (0.983-0.989) |
| Precision (95% CI) | 0.978 (0.972-0.983) | 0.979 (0.975-0.984) |
| Recall (95% CI) | 0.986 (0.982-0.989) | 0.986 (0.982-0.989) |
| *F*_1_-score (95% CI) | 0.982 (0.977-0.986) | 0.983 (0.979-0.986) |
| ROC AUC^a^ (95% CI) | 0.998 (0.997-0.999) | 0.998 (0.997-0.999) |

^a^ ROC AUC: area under the receiver operating characteristic curve.

### B.1.2 Prior-model benchmark

To further benchmark our Orchestrate-Contrast model against existing BOA-Contrast [1] and FALCON [2] on both the internal framework evaluation dataset and the external datasets, with radiographer-derived reference standards. BOA-Contrast used calculated HU features from relevant body structures produced by the BOA segmentation framework [3] and differentiated contrast phases for both intravenous and gastrointestinal contrast [1]. In contrast, our model and FALCON perform a more focused binary classification of the presence or absence of intravenous contrast using different input formats. FALCON used a single central 2D axial CT slice as input [2], whereas our model used 4 slices combined into a single PNG image. The average inference time was approximately 2 min per series for BOA-Contrast, compared with approximately 7 ms per image for our model. We could not directly measure the average inference time for FALCON because it is implemented as a desktop application. However, according to the original publication, inference is completed within seconds per CT scan [2].

Both BOA-Contrast and Orchestrate-Contrast successfully processed all internal (n = 738) and external (n = 113) series. In contrast, FALCON processed only a subset of the dataset. Among internal series, 42 could not be processed: 33 returned a “list index out of range” error, and 9 returned a “not enough DICOM slices in the directory” error. Therefore, only 696 internal series were included in the FALCON performance analysis. In the external dataset, 23 series could not be processed because of a “list index out of range” error, and the remaining 90 were included in this analysis. The weighted and macro F1 scores for all models were reported in Table B1.2.

Table B1.2 Comparative Performance of Orchestrate-Contrast, BOA-Contrast, and FALCON.

|  | **Data** | **Orchestrate – Contrast** | **BOA – Contrast** | **FALCON**^c^ |
| --- | --- | --- | --- | --- |
| Weighted *F*_1_-score (95% CI) | Internal^a^ | 0.989  (0.981-0.996) | 0.927  (0.904-0.946) | 0.871  (0.842-0.897) |
| Macro–*F*_1_-score (95% CI) | Internal | 0.983  (0.970-0.994) | 0.876  (0.842-0.906) | 0.790  (0.750-0.829) |
| Weighted *F*_1_-score (95% CI) | External^b^ | 0.946  (0.909-0.979) | 0.889  (0.820-0.947) | 0.867  (0.774-0.938) |
| Macro–*F*_1_-score (95% CI) | External | 0.777  (0.582-0.905) | 0.470  (0.452-0.484) | 0.464  (0.441-0.483) |

^a^ “Internal” indicates the data from the internal framework evaluation dataset.

^b^ “External” indicates the data from the external dataset.

^c^ For FALCON, the F1 scores were calculated only using the successfully processed cases.

The results presented in Table B1.2 revealed several interesting findings. Both the Orchestrate-Contrast and BOA-Contrast achieved relatively higher performance. Figure B1 visualized representative cases in which the predictions of three models differed.

Figure B1. Representative cases of disagreement among the Orchestrate-Contrast, BOA-Contrast, and FALCON.

For instance, in Figure B1(a), with reference standard native, FALCON classified the scan as native with a confidence score of 0.971, and Orchestrate-Contrast also classified it as native. In contrast, BOA-Contrast assigned probabilities of 0.436 and 0.503 to native and urographic phases, respectively. In Figure B1(b), with reference standard native, only Orchestrate-Contrast classified the scan as contrast-enhanced, whereas FALCON classified it as native with a confidence score of 1.000, and BOA-Contrast assigned a probability of 0.958 to the native. In Figure B1(c), with reference standard contrast-enhanced, both Orchestrate-Contrast and BOA-Contrast classified the scan as contrast-enhanced, with BOA-Contrast assigning a probability of 0.999 to the arterial phase, but FALCON classified the scan as native with a confidence score of 1.000.

To further characterize prediction consistency across models, independent of the reference standard, we also assessed pairwise agreement among the three models using Cohen’s Kappa. The highest agreement was observed between our model and the BOA-Contrast (Cohen κ=0.806, 95% CI 0.757-0.848), followed by the BOA-Contrast and the FALCON (Cohen κ=0.773, 95% CI 0.720-0.824). Our model and FALCON showed the lowest agreement (Cohen κ=0.667, 95% CI 0.603-0.725). The incomplete agreement among the three model predictions, together with the example cases observed in Figure B1, further highlights the complexity of contrast-enhancement classification.

### B.1.3 Sensitivity Analysis of Slice-Sampling Strategy

To assess the potential influence of slice sampling, a sensitivity analysis was performed by varying the inter-slice offset while maintaining 4 slices per CT series combined as a single PNG image as input. We kept the trained Orchestrate-BOA model checkpoint and all other settings unchanged. The classification performance was compared on the internal framework evaluation dataset across different slice-spacing configurations using macro and weighted F1 scores (Table B1.3).

Table B1.3 Comparative Performance of different slice sampling strategies

| Slice Sampling Strategy | Macro–*F*_1_-score (95% CI) | Weighted *F*_1_-score (95% CI) |
| --- | --- | --- |
| Offset = 2 | 0.970 (0.953-0.985) | 0.981 (0.970-0.991) |
| Offset = 5 | 0.983 (0.970-0.994) | 0.989 (0.981-0.996) |
| Offset = 10 | 0.958 (0.939-0.976) | 0.974 (0.963-0.985) |

Classification performance varied only slightly across the three settings, with the original offset of 5 achieving slightly higher performance than offsets of 2 and 10. These findings suggest that the model is relatively robust to variations in slice spacing.

## B.2 Reference Standard Analysis

Inter-rater agreement was consistently high across all classification tasks (Table B2). All Fleiss κ exceeded 0.930, and all pairwise Cohen κ values exceeded 0.906, indicating strong agreement among the three human experts.

Table B2: Inter-rater agreement among three radiographers for reference standard.

| **Model** | **Measure** | **Estimate (95% CI)** | **n** |
| --- | --- | --- | --- |
| **Reconstruction Kernel** |  |  |  |
|  | Fleiss κ | 0.931 (0.910-0.950) | 851^a^ |
|  | Cohen κ(R1, R2) | 0.919 (0.891-0.943) | 851 |
|  | Cohen κ(R1, R3) | 0.907 (0.878-0.933) | 851 |
|  | Cohen κ(R2, R3) | 0.969 (0.949-0.983) | 851 |
| **Body Contrast Enhancement** |  |  |  |
|  | Fleiss κ | 0.936 (0.915-0.956) | 851 |
|  | Cohen κ(R1, R2) | 0.933 (0.907-0.958) | 851 |
|  | Cohen κ(R1, R3) | 0.908 (0.876-0.936) | 851 |
|  | Cohen κ(R2, R3) | 0.969 (0.949-0.986) | 851 |
| **Brain Contrast Enhancement** |  |  |  |
|  | Fleiss κ | 0.958 (0.918-0.991) | 225^b^ |
|  | Cohen κ(R1, R2) | 0.949 (0.894-0.988) | 225 |
|  | Cohen κ(R1, R3) | 0.961 (0.911-1.000) | 225 |
|  | Cohen κ(R2, R3) | 0.962 (0.914-1.000) | 225 |

^a^ The total of 851 series comprised 738 internal series and 113 external series.

^b^ The 225 series comprised only internal data, as external data did not include head structure.

## B.3 Orchestrate Error Analysis

For the View Position classifier, 3 external non-lateral topograms acquired using Philips scanners were misclassified as lateral, and 3 external lateral topograms acquired using Toshiba scanners (now Canon Medical Systems) were classified as non-lateral. Figure B2 shows visualization examples of the internal input topogram data and the error cases from external data from both scanners. To enable a direct visual comparison of input image appearance, we applied no additional contrast or intensity adjustments for visualization.

Figure B2. Visualization examples of internal input data and external input cases with incorrect model predictions.

For the Reconstruction Kernel classifier, we observed no misclassifications in the external dataset. Examination of the misclassified cases in the internal framework evaluation dataset demonstrated that, for the reference standard, the three radiographers did not reach complete agreement on any of these cases, with at least one radiographer assigning a different label from the others. Furthermore, the reconstruction kernel parameters for these cases, such as Bl57 and Br38, were close to the boundary between the predefined soft and hard kernels. The findings suggested that the misclassifications were primarily attributable to the borderline of these cases, which made their classification inherently challenging for the model.

For the Body Contrast Enhancement classifier, the observed misclassifications may be attributed to several factors. First, as discussed in the previous Section B.1.2, contrast-enhancement classification is inherently challenging. Second, the model used only 4 selected slices combined into a single PNG image as input. Therefore, these selected slices may not capture regions where intravenous contrast enhancement is most apparent, limiting the information available to the model. Although slices without obvious enhancement in a contrast-enhanced CT scan may still differ from those in a non-contrast scan, distinguishing such cases remained challenging. Finally, the slightly lower performance observed in the external cohort may reflect differences in data characteristics, where some external CT scans contained gastrointestinal contrast, which was not the target of the intravenous contrast classifier and may have introduced additional visual features that complicated classification.

Contrast enhancement classification in brain CT may be more challenging than in abdominal or thoracic CT because current iodinated contrast media do not readily cross the intact blood-brain barrier, resulting in only a slight increase in enhancement of normal cerebral parenchyma [4]. This may partly explain the lower performance in Brain Contrast Enhancement compared to the Body Contrast Enhancement. In addition, the model relies on four selected slices to determine the contrast status. This may not always capture relevant enhancement features, particularly when enhancement is subtle or not readily apparent in the selected slices, which can partly explain why most misclassifications involved contrast-enhanced scans being incorrectly classified as native.

## References

1. Baldini G, Hosch R, Schmidt CS, Borys K, Kroll L, Koitka S, Haubold P, Pelka O, Nensa F, Haubold J. Addressing the Contrast Media Recognition Challenge: A Fully Automated Machine Learning Approach for Predicting Contrast Phases in CT Imaging. Invest Radiol 2024 Sept;59(9):635–645. doi: 10.1097/RLI.0000000000001071
2. Westphal JA, Kaess P, Mantz L, Barnawi RA, Saidi S, Fabritius MP, Ziller A, Kaissis G, Rueckert D, Fintelmann FJ. Open-Access Fully Automated Intravenous Contrast Detection and Body Part Classification for Computed Tomography Scans: The FALCON Model. J Imaging Inform Med 2026 Feb 26; PMID:41749033
3. Haubold J, Baldini G, Parmar V, Schaarschmidt BM, Koitka S, Kroll L, van Landeghem N, Umutlu L, Forsting M, Nensa F, Hosch R. BOA: A CT-Based Body and Organ Analysis for Radiologists at the Point of Care. Invest Radiol 2024 June 1;59(6):433–441. PMID:37994150
4. Sage MR, Wilson AJ, Scroop R. Contrast media and the brain. The basis of computed tomography and magnetic resonance imaging enhancement: A review. Australas Radiol 2000 May;44(2):133–142. doi: 10.1046/j.1440-1673.2000.00797.x
