# Supplementary material for: A Multi-Model, Pixel-Native Framework for Automated Computed Tomography Series Labeling and Characterization: Proof-of-Concept Study
Source: JMIR Med Inform. 2026 Sep 29;14:e93018. doi: 10.2196/93018 (PMC13662436; doi:10.2196/93018)
Supplement: Supplementary file 3 [file medinform_v14i1e93018_app3.docx]

# Potential Fast Healthcare Interoperability Resources (FHIR) profile

To facilitate standardized data exchange and interoperability, a potential FHIR profile, OrchestrateObservation, was developed to encapsulate all Orchestrate results associated with CT metadata within a unified FHIR Observation resource. This profile adheres to the HL7 FHIR R4 specification [1], ensuring compliance with established healthcare interoperability standards and seamless integration with clinical information systems.

Figure C. Relational Schema of the OrchestrateObservation FHIR profile. This diagram demonstrates the reference architecture between the OrchestrateObservation profile and associated FHIR resources. The OrchestrateObservation requires a reference to the Patient resource via the subject element, indicating which patient the observation belongs to. It has references to ImagingStudy via “derivedFrom”, which links to the source CT series, and to Procedure via “partOf”, indicating the clinical procedure context.

The Observation resource was selected as the parent structure because it supports multiparameter measurements through hierarchical component elements. Each model output, along with the associated DICOM metadata, was represented as a separate component within the resource. The example values of defined components are shown in Table C.

Table C. Component structure and representative examples.

| **Component** | **Type** | **Code** | **Display** | **Value** | **units** | **Description** |
| --- | --- | --- | --- | --- | --- | --- |
| studyUID | string | - |  | 1.2.3….10 | - | DICOM Study Instance UID |
| seriesUID | string | - |  | 1.2.3….01 | - | DICOM Series Instance UID |
| sliceThickness | Quantity | - |  | 1 | mm | Reconstructed slice thickness |
| reconstructionKernel | string | - |  | SK | - | CT reconstruction kernel |
| bodycontrastPhase | string | - |  | native | - | Body CT contrast enhancement information |
| braincontrastPhase | string | - |  | native | - | Brain CT contrast enhancement information |
| bodyLandmark | CodeableConcept (from BodyLandmarkVS) +Quantity | 39607008 | Lung | 30 | % | Defined anatomical landmark with a percentage value |
| bodyRegion | CodeableConcept (from BodyRegionVS) +Quantity | 281825004 | Thoracic Region | 32 | % | Defined anatomical regions with a percentage value |

Anatomical coverage is ensured through body landmark and body region codes within the observation components, which are constrained to the BodyLandmarkVS and BodyRegionVS value sets, respectively. The value sets reference Systematized Nomenclature of Medicine - Clinical Terms (SNOMED CT) [2] body structure codes from their code systems, ensuring terminological and semantic interoperability.

## References

1. Index - FHIR v4.0.1. Available from: https://hl7.org/fhir/R4/ [accessed 2026-09-16]
2. What is SNOMED CT. SNOMED Int. Available from: https://www.snomed.org/what-is-snomed-ct [accessed 2026-01-14]
