# Supplementary material for: A Multi-Model, Pixel-Native Framework for Automated Computed Tomography Series Labeling and Characterization: Proof-of-Concept Study
Source: JMIR Med Inform. 2026 Sep 29;14:e93018. doi: 10.2196/93018 (PMC13662436; doi:10.2196/93018)
Supplement: Supplementary file 4 [file medinform_v14i1e93018_app4.docx]

# Head region exclusion for anonymization

The head portion of a CT series that contains identifiable facial anatomy can be automatically excluded, enabling anonymization without altering the remaining imaging content.

For the CT series, the head position predicted from the topogram is mapped back onto the CT series data. This enables identification of the slice range containing craniofacial anatomy by quantifying the proportion of the slice range occupied by the head relative to the entire scan length. The algorithm then determines whether the CT scan was obtained in the cranial-to-caudal or caudal-to-cranial orientation. Based on this, the pipeline removes the appropriate portion of the series, either from the first slices or the final slices, corresponding to the predicted head region. After the facial-containing slices have been removed, metadata relevant to the anonymization process, including the scan orientation, slice count, and mid-coronal images from both original and anonymized series, are stored in the head_deid table within the final database. An example of the original and anonymized CT series is shown in Figure D.

Figure D: Overview of the Head Anonymization results. Mid-coronal slices from the original CT series (left) and post-processed anonymized series (right). The craniofacial region has been excised according to acquisition orientation.

To assess automated head anonymization performance, 200 CT series containing craniofacial anatomy were randomly selected for manual review. The experienced radiographer evaluated the anonymized datasets using a 5-point Likert scale, with particular attention to whether identifiable head structures had been removed. Among these, 192 CT series exhibited complete removal of the head region, 5 CT series were rated as good performance, and 3 were judged to be acceptable.

Importantly, this application is not limited to the head. The same approach can be generalized to isolate or exclude other anatomical regions. For example, clinicians interested only in abdominal slices from the whole-body CT series could use the pipeline to isolate or remove specific anatomical regions, enabling flexible dataset tailoring while preserving patient privacy.
